# EAT-UpTF: Enrichment Analysis Tool for Upstream Transcription Factors of a Group of Plant Genes

**DOI:** 10.3389/fgene.2020.566569

**Published:** 2020-09-11

**Authors:** Sangrea Shim, Pil Joon Seo

**Affiliations:** ^1^Department of Chemistry, Seoul National University, Seoul, South Korea; ^2^Plant Genomics and Breeding Institute, Seoul National University, Seoul, South Korea; ^3^Research Institute of Basic Sciences, Seoul National University, Seoul, South Korea

**Keywords:** transcription factor, *cis*-elements, plant, *Arabidopsis*, DAP-seq

## Abstract

EAT-UpTF (Enrichment Analysis Tool for Upstream Transcription Factors of a group of plant genes) is an open-source Python script that analyzes the enrichment of upstream transcription factors (TFs) in a group of genes-of-interest (GOIs). EAT-UpTF utilizes genome-wide lists of TF-target genes generated by DNA affinity purification followed by sequencing (DAP-seq) or chromatin immunoprecipitation followed by sequencing (ChIP-seq). Unlike previous methods based on the two-step prediction of *cis*-motifs and DNA-element-binding TFs, our EAT-UpTF analysis enabled a one-step identification of enriched upstream TFs in a set of GOIs using lists of empirically determined TF-target genes. The tool is designed particularly for plant researches, due to the lack of analytic tools for upstream TF enrichment, and available at https://github.com/sangreashim/EAT-UpTF and http://chromatindynamics.snu.ac.kr:8080/EatupTF.

## Introduction

The rapid development of high-throughput technologies such as RNA sequencing (RNA-seq), DNA affinity purification followed by sequencing (DAP-seq), and chromatin immunoprecipitation followed by sequencing (ChIP-seq) has led to an explosion in the availability of sequence data. The high-throughput analyses produce lists of genes that are under a particular regulation. When such lists are generated, researchers usually try to understand the biological implications of groups of genes-of-interest (GOIs). To this end, routine follow-up studies typically include gene ontology (GO) enrichment analyses (Maere et al., 2005; Huang et al., 2009) and Kyoto Encyclopedia of Genes and Genomes (KEGG) mapping (Kanehisa and Goto, 2000). In addition, transcription factor (TF) prediction analyses (Kreft et al., 2017; Kulkarni et al., 2018) can be performed to identify consensus upstream regulators of a subset of GOIs, giving a biological insight into the integrated role of the genes under specific conditions. Furthermore, comprehensive identification of TF binding sites and cognate TFs can be used to characterize regulatory networks containing GOIs.

Several bioinformatics tools have been developed to predict upstream TFs. The *cis*-element sequences that are commonly conserved in sets of input query genes can be identified using *ab initio* motif enrichment algorithms such as MEME (Bailey et al., 2009). The identified consensus sequences can be further analyzed to compare enrichment of TF candidates to the consensus binding motifs provided by databases of experimentally validated TF binding sites, such as JASPAR (Khan et al., 2018) and TRANSFAC (Matys et al., 2003). Recently, accumulating data have enabled that position weight matrix (PWM)-based enrichment methods solely cover a wide range of upstream TF prediction. This theoretical basis has been implemented in various upstream TF prediction tools, such as TFEA.ChIP, oPOSSUM, and PlantRegMap (Ho Sui et al., 2005; Puente-Santamaria et al., 2019; Tian et al., 2020). However, this approach occasionally produces a considerable number of false positives due to short and degenerate nature of TF-binding sites (Kreft et al., 2017). In addition, this method is complicated by the fact that TFs can sometimes bind to gene sequences that differ from their consensus binding sites, and that several TFs undergo protein–protein interactions that enable them to recognize additional DNA sequence motifs. Overall, it is clear that a simplified and realistic prediction of TFs controlling a group of GOIs is necessary to generate a confident conclusion.

In this regard, several bioinformatics tools implementing TF enrichment analysis have been developed using ChIP-seq datasets (Zambelli et al., 2012; Auerbach et al., 2013; Zheng et al., 2019). However, these tools are applicable mainly to animal systems, and no codes have been released to analyze enriched upstream TFs for other species. Based on explosive accumulation of plant DAP-seq and ChIP-seq data, there are growing needs to integrate the NGS data and use them to retrieve upstream TFs in plant researches. Notably, O’Malley and colleagues adapted the innovative DAP-seq method and have successfully produced a genome-wide collection of target genes for 349 TFs in *Arabidopsis thaliana* (O’Malley et al., 2016). In this study, we have developed the “Enrichment Analysis Tool for Upstream Transcription Factors of a group of plant genes” (EAT-UpTF) tool to provide upstream TF enrichment analysis (Shim and Seo, 2020). As a proof of concept, we combined it with the *Arabidopsis* DAP-seq database to analyze the enrichment of upstream TFs in a group of *Arabidopsis* GOIs. We found that EAT-UpTF was able to robustly evaluate the over-representation of experimentally validated upstream TFs binding to a group of GOIs without the prediction of *cis*-motifs.

## Methods

**FIGURE 1:**
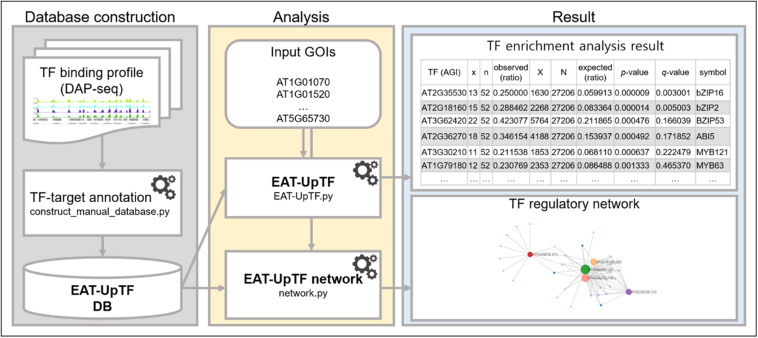
Workflow of EAT-UpTF. Manual database can be constructed based on binding profiles of multiple TFs generated by DAP-seq and ChIP-seq using manual database construction module (construct_manual_database.py). When a set of genes of interest (GOIs) is input along with database, EAT-UpTF performs enrichment analysis and shows the overrepresented upstream TFs for the GOIs. Network construction module (network.py) also visualizes regulatory networks of TFs and their target genes.

High-throughput sequencing analyses typically produce sets of GOIs that require further analyses to evaluate their biological implication and underlying regulatory mechanisms. EAT-UpTF is linked to a DAP-seq database (Plant Cistrome database^1^) that provides a list of TF-target genes (locus IDs). When a set of GOIs is input in the form of locus IDs, EAT-UpTF identifies the TF targets and compares their relative enrichment in the list of GOIs with that in the total genomic genes. As a result, target genes of certain TFs, which are enriched (over-represented) in the set of GOIs can be identified as a major upstream regulators of the gene group (Figure 1). To examine the statistical significance of over-representation, the SciPy module (Oliphant, 2007) is used to perform hypergeometric and binomial tests, which differ in that the binomial test considers replacement whereas the hypergeometric test does not. These two tests are used to compare the occurrence of *x* genes (a subset of TF-target genes) among *n* genes (GOIs) with that of *X* genes (total TF-target genes) among *N* genes (total reference genes). Comparisons with relatively large differences (*x/n – X/N*) can then be considered to identify upstream TFs that may play a particular role in regulating at least a subset of GOIs.

For the initial validation of EAT-UpTF, we used the DAP-seq *Arabidopsis* database, which lists the target genes of a vast majority of *Arabidopsis* TFs (∼349). Since EAT-UpTF performs enrichment analyses for hundreds of TFs simultaneously, a *post hoc* test should be applied to counteract the type I errors (false positives) originating from multiple testing. A number of *post hoc* analyses can be used to compensate for the increase in the false positive rate caused by multiple tests. The most widely used method is the family-wise error rate (FWER) correction, named after Carlo Emilio Bonferroni. The Bonferroni correction tests individual hypotheses at a significance level of *a*/*m*, where *a* is the desirable alpha level and *m* is the number of tests performed (Bonferroni et al., 1936; Dunn, 1961). This correction method is considered conservative when a large number of tests are conducted, but was likely appropriate in our analysis because the multiple hypothesis tests were limited to several hundreds of TFs. Another *post hoc* analysis option is the false discovery rate (FDR) correction described by Benjamini and Hochberg (1995). The Benjamini-Hochberg FDR correction tests hypotheses at a significance level of *ka/m*, where *a* is the desirable alpha level, *m* is the number of tests performed, and *k* is the rank of the *p*-value of the hypothesis. These two most popular *post hoc* analyses have been implemented in the current version of EAT-UpTF using the Statsmodels module of Python (Seabold and Perktold, 2010).

## Results and Discussion

To validate the relevance of EAT-UpTF, we input a gene set bound by the LATE ELONGATED HYPOCOTYL (LHY) TF in *Arabidopsis*, which was identified via a ChIP-seq analysis (Adams et al., 2018). EAT-UpTF identified LHY as being an over-represented upstream TF in the test set. Specifically, 71.6% of the input genes were retrieved to be bound by LHY (Table 1) and LHY was identified as one of the top five enriched TFs in the test set (Table 1). The mismatch between the EAT-UpTF output and the ChIP-seq data might be related to the fact that DAP-seq is generally more stringent than ChIP-seq. Typically, DAP-seq produces a rigorous gene set and usually identifies a smaller number of TF-target genes than ChIP-seq. Indeed, all of the LHY-target genes identified by DAP-seq were included in the list of LHY-target genes identified by ChIP-seq analysis.

**TABLE 1:** Summary statistics of the upstream transcription factor (TF) enrichment analysis for the *Arabidopsis* gene set bound by LHY (Adams et al., 2018).

**TF ID (AGI ID)**	***x*^a^**	***n*^b^**	**Observed (%)**	***X*^c^**	***N*^d^**	**Expected (%)**	***p*-Value**	**Corrected *p*-value^e^**	**Gene symbols**	**Gene names**
AT5G02840	287	722	39.8	4,110	27,206	15.1	5.84 × 10^–60^	2.04 × 10^–57^	*LCL1*	LHY/CCA1-LIKE 1
AT3G09600	426	722	59.0	8,276	27,206	30.4	2.59 × 10^–58^	4.52 × 10^–56^	*RVE8*, *LCL5*	LHY-CCA1-LIKE5, REVEILLE 8
AT3G56850	275	722	38.1	3,936	27,206	14.5	6.43 × 10^–57^	7.48 × 10^–55^	*AREB3*, *DPBF3*	ABA-RESPONSIVE ELEMENT BINDING PROTEIN 3
AT2G46270	318	722	44.0	5,255	27,206	19.3	2.09 × 10^–53^	1.82 × 10^–51^	*GBF3*	G-BOX BINDING FACTOR 3
AT1G01060	517	722	71.6	11,896	27,206	43.7	3.01 × 10^–53^	2.10 × 10^–51^	*LHY*	LATE ELONGATED HYPOCOTYL
AT2G36270	274	722	38.0	4,188	27,206	15.4	8.13 × 10^–51^	4.73 × 10^–49^	*ABI5*, *GIA1*	GROWTH-INSENSITIVITY TO ABA 1, ABA INSENSITIVE 5
AT3G62420	327	722	45.3	5,764	27,206	21.2	7.54 × 10^–49^	3.76 × 10^–47^	*BZIP53*	BASIC REGION/LEUCINE ZIPPER MOTIF 53
AT1G18330	619	722	85.7	16,878	27,206	62.0	3.63 × 10^–46^	1.58 × 10^–44^	*EPR1*, *RVE7*	EARLY-PHYTOCHROME-RESPONSIVE 1, REVEILLE 7
AT5G17300	585	722	81.0	15,403	27,206	56.6	6.78 × 10^–45^	2.63 × 10^–43^	*RVE1*	REVEILLE 1
AT1G32150	357	722	49.4	6,979	27,206	25.7	6.05 × 10^–44^	2.11 × 10^–42^	*bZIP68*	BASIC REGION/LEUCINE ZIPPER TRANSCRIPTION FACTOR 68
AT4G34590	381	722	52.8	7,781	27,206	28.6	1.94 × 10^–43^	6.15 × 10^–42^	*GBF6*, *BZIP11*, *ATB2*	ARABIDOPSIS THALIANA BASIC LEUCINE-ZIPPER 11, G-BOX BINDING FACTOR 6
AT5G52660	224	722	31.0	3,280	27,206	12.1	6.20 × 10^–43^	1.80 × 10^–41^		
AT5G15830	336	722	46.5	6,440	27,206	23.7	2.94 × 10^–42^	7.88 × 10^–41^	*bZIP3*	BASIC LEUCINE-ZIPPER 3
AT2G18160	178	722	24.7	2,268	27,206	8.3	4.60 × 10^–41^	1.15 × 10^–39^	*GBF5*, *bZIP2*, *ATBZIP2*, *FTM3*	BASIC LEUCINE-ZIPPER 2, FLORAL TRANSITION AT THE MERISTEM3, G-BOX BINDING FACTOR 5
AT4G01280	339	722	47.0	6,654	27,206	24.5	1.88 × 10^–40^	4.38 × 10^–39^		
AT3G10113	579	722	80.2	15,664	27,206	57.6	6.91 × 10^–39^	1.51 × 10^–37^		
AT1G45249	165	722	22.9	2,112	27,206	7.8	1.45 × 10^–37^	2.98 × 10^–36^	*ABF2*, *AREB1*	ABSCISIC ACID RESPONSIVE ELEMENTS-BINDING PROTEIN 1, ABSCISIC ACID RESPONSIVE ELEMENTS-BINDING FACTOR 2
AT3G10800	132	722	18.3	1,469	27,206	5.4	7.86 × 10^–36^	1.52 × 10^–34^	*BZIP28*	
AT4G36780	269	722	37.3	4,944	27,206	18.2	1.02 × 10^–34^	1.88 × 10^–33^	*BEH2*	BES1/BZR1 HOMOLOG 2
AT2G35530	137	722	19.0	1,630	27,206	6.0	3.89 × 10^–34^	6.79 × 10^–33^	*bZIP16*	BASIC REGION/LEUCINE ZIPPER TRANSCRIPTION FACTOR 16

*^*a*^The number of genes bound by the specific TF in the test set. ^*b*^The number of genes in the test set. ^*c*^The number of genes bound by the specific TF in the reference set. ^*d*^The number of genes in the reference set. ^*e*^The *p*-value after Bonferroni or Benjamini-Hochberg correction.*

We also compared EAT-UpTF analysis to a conventional motif enrichment analysis for a similar purpose. DREME, a motif enrichment algorithm of MEME suite (Bailey et al., 2009), identified 33 conserved sequence motifs that can be bound by 157 TFs (Supplementary Table 1). While the LHY transcription factor was predicted, which could bind to two motifs, AAATATCK and GATATTTW (Supplementary Table 1), a vast number of additional *cis*-elements, which are not related to LHY, were also suggested. These results indicate that a motif enrichment analysis possibly produces a considerable number of false positives, but EAT-UpTF enables to suggest realistic upstream TFs.

To ensure whether the EAT-UpTF analysis is relevant with less stringent data set, we input DEGs in *ccal lhy* double mutant relative to wild type identified by RNA-seq (Kamioka et al., 2016). Again, EAT-UpTF identified LHY as an over-represented upstream TF for the input gene set (Table 2). Since CCA1 and LHY are transcriptional repressors (Kamioka et al., 2016), a significant portion of up-regulated genes in *cca1 lhy* was supposed to be direct targets of CCA1 and LHY. Indeed, EAT-UpTF predicted LHY as a top ranked TF for up-regulated genes in *cca1 lhy* double mutant (Supplementary Table 2), whereas LHY was excluded but other bZIP TFs were identified to be bound to down-regulated genes in *cca1 lhy* (Supplementary Table 3).

**TABLE 2:** Summary statistics of enriched upstream TFs for differentially expressed genes (DEGs) in *cca1lhy* double mutant (Kamioka et al., 2016).

**TF ID (AGI ID)**	***x*^a^**	***n*^b^**	**Observed (%)**	***X*^c^**	***N*^d^**	**Expected (%)**	***p*-Value**	**Corrected *p*-alue^e^**	**Gene symbols**	**Gene names**
AT5G02840	267	824	32.4	4,110	27,206	15.1	9.65 × 10^–37^	3.37 × 10^–34^	*LCL1*	LHY/CCA1-LIKE 1
AT4G01280	329	824	39.9	6,654	27,206	24.5	1.75 × 10^–23^	3.05 × 10^–21^		
AT5G52660	196	824	23.8	3,280	27,206	12.1	1.71 × 10^–21^	1.98 × 10^–19^		
AT3G09600	374	824	45.4	8,276	27,206	30.4	3.27 × 10^–20^	2.85 × 10^–18^	*LCL5, RVE8*	LHY-CCA1-LIKE5, REVEILLE 8
AT1G01060	479	824	58.1	11,896	27,206	43.7	2.47 × 10^–17^	1.72 × 10^–15^	*LHY1, LHY*	LATE ELONGATED HYPOCOTYL 1, LATE ELONGATED HYPOCOTYL
AT3G62420	275	824	33.4	5,764	27,206	21.2	1.20 × 10^–16^	6.99 × 10^–15^	*BZIP53*	BASIC REGION/LEUCINE ZIPPER MOTIF 53
AT4G34590	344	824	41.7	7,781	27,206	28.6	1.75 × 10^–16^	8.70 × 10^–15^	*BZIP11, GBF6, ATB2*	G-BOX BINDING FACTOR 6, NA, ARABIDOPSIS THALIANA BASIC LEUCINE-ZIPPER 11
AT2G46270	250	824	30.3	5,255	27,206	19.3	9.54 × 10^–15^	4.16 × 10^–13^	*GBF3*	G-BOX BINDING FACTOR 3
AT1G18330	610	824	74.0	16,878	27,206	62.0	9.71 × 10^–14^	3.76 × 10^–12^	*RVE7, EPR1*	REVEILLE 7, EARLY-PHYTOCHROME-RESPONSIVE1
AT3G56850	194	824	23.5	3,936	27,206	14.5	1.39 × 10^–12^	4.86 × 10^–11^	*AREB3, DPBF3*	ABA-RESPONSIVE ELEMENT BINDING PROTEIN 3
AT5G17300	560	824	68.0	15,403	27,206	56.6	8.36 × 10^–12^	2.65 × 10^–10^	*RVE1*	REVEILLE 1
AT1G32150	297	824	36.0	6,979	27,206	25.7	1.37 × 10^–11^	3.97 × 10^–10^	*bZIP68*,	BASIC REGION/LEUCINE ZIPPER TRANSCRIPTION FACTOR 68
AT2G18160	126	824	15.3	2,268	27,206	8.3	1.78 × 10^–11^	4.77 × 10^–10^	*GBF5, bZIP2, FTM3*	BASIC LEUCINE-ZIPPER 2, G-BOX BINDING FACTOR 5, FLORAL TRANSITION AT THE MERISTEM 3
AT5G15830	278	824	33.7	6,440	27,206	23.7	2.02 × 10^–11^	5.03 × 10^–10^	*bZIP3*	BASIC LEUCINE-ZIPPER 3
AT1G45249	119	824	14.4	2,112	27,206	7.8	2.95 × 10^–11^	6.87 × 10^–10^	*AREB1, ABF2*	ABSCISIC ACID RESPONSIVE ELEMENTS-BINDING FACTOR 2, ABSCISIC ACID RESPONSIVE ELEMENTS-BINDING PROTEIN 1
AT2G36270	198	824	24.0	4,188	27,206	15.4	3.38 × 10^–11^	7.38 × 10^–10^	*GIA1, ABI5*	GROWTH-INSENSITIVITY TO ABA 1, ABA INSENSITIVE 5
AT2G35530	97	824	11.8	1,630	27,206	6.0	1.44 × 10^–10^	2.96 × 10^–9^	*bZIP16*,	BASIC REGION/LEUCINE ZIPPER TRANSCRIPTION FACTOR 16
AT3G10113	559	824	67.8	15,664	27,206	57.6	5.25 × 10^–10^	1.02 × 10^–8^		
AT1G75390	127	824	15.4	2,485	27,206	9.1	2.97 × 10^–9^	5.46 × 10^–8^	*bZIP44*	BASIC LEUCINE-ZIPPER 44
AT3G10800	82	824	10.0	1,469	27,206	5.4	7.24 × 10^–8^	1.26 × 10^–6^	*BZIP28*	

*^*a*^The number of genes bound by the specific TF in the test set. ^*b*^The number of genes in the test set. ^*c*^The number of genes bound by the specific TF in the reference set. ^*d*^The number of genes in the reference set. ^*e*^The *p*-value after Bonferroni or Benjamini-Hochberg correction.*

In addition, we further examined the relevance of EAT-UpTF in upstream TF enrichment analysis using unoptimized datasets. Genes up-regulated and down-regulated in root tissues upon 1 μM IAA treatment for 6 h (Omelyanchuk et al., 2017) were used as input queries. As for the up-regulated genes, EAT-UpTF identified LATERAL ORGAN BOUNDARIES DOMAIN 19 (LBD19), LBD18 and LBD16 as upstream regulators, which are involved in auxin-dependent lateral root emergence (Feng et al., 2012) (Table 3). Meanwhile, BASIC REGION/LEUCINE ZIPPER MOTIF 53 (bZIP53) and bZIP11, which negatively regulate adventitious root formation and primary root growth in an auxin-dependent pathway (Weiste et al., 2017; Zhang et al., 2020), were retrieved as overrepresented upstream TFs for the IAA-repressed genes (Table 4). Overall, the EAT-UpTF analysis reliably identified upstream TFs for a group of GOIs. Although our study mainly focused on the enriched upstream TFs for input query genes, which provides essential interpretation of the GOIs in the context of biological pathways and networks, we cannot rule out that TFs regulating a subset of input genes are also sometimes important for estimating biological functions of GOIs, independently of statistical enrichment. Thus, EAT-UpTF can also be used for profiling all possible upstream TFs that potentially regulate GOIs.

**TABLE 3:** Summary statistics of enriched upstream TFs for up-regulated genes in *Arabidopsis* roots upon 1 μM IAA treatment for 6 h (Omelyanchuk et al., 2017).

**TF ID (AGI ID)**	***x*^a^**	***n*^b^**	**Observed (%)**	***X*^c^**	***N*^d^**	**Expected (%)**	***p*-Value**	**Corrected *p*-value^e^**	**Gene symbols**	**Gene names**
AT1G72740	172	789	21.8	2,924	27,206	10.7	5.21 × 10^–^^20^	1.82 × 10^–17^		
AT2G45410	303	789	38.4	6,835	27,206	25.1	4.78 × 10^–17^	1.67 × 10^–14^	*LBD19*	LOB DOMAIN-CONTAINING PROTEIN 19
AT2G45420	215	789	27.2	4,503	27,206	16.6	1.11 × 10^–14^	3.88 × 10^–12^	*LBD18*	LOB DOMAIN-CONTAINING PROTEIN 18
AT5G59430	49	789	6.2	563	27,206	2.1	9.88 × 10^–12^	3.45 × 10^–9^	*TRP1*,	TELOMERIC REPEAT BINDING PROTEIN 1
AT3G46590	33	789	4.2	363	27,206	1.3	8.56 × 10^–9^	2.99 × 10^–6^	*TRP2, TRFL1, ATTRP2*	TRF-LIKE 1
AT5G67580	221	789	28.0	5,446	27,206	20.0	2.85 × 10^–8^	9.94 × 10^–6^	*TRB2, TBP3*	TELOMERE-BINDING PROTEIN 3, TELOMERE REPEAT BINDING FACTOR 2
AT1G34670	136	789	17.2	3,086	27,206	11.3	3.89 × 10^–7^	1.36 × 10^–4^	*MYB93*	MYB DOMAIN PROTEIN 93
AT4G32730	269	789	34.1	7,322	27,206	26.9	3.87 × 10^–6^	1.35 × 10^–3^	*MYB3R1, PC-MYB1*	MYB DOMAIN PROTEIN 3R1, C-MYB-LIKE TRANSCRIPTION FACTOR 3R-1
AT5G11510	83	789	10.5	1,732	27,206	6.4	4.80 × 10^–6^	1.68 × 10^–3^	*AtMYB3R4*	MYB DOMAIN PROTEIN 3R4
AT2G02820	249	789	31.6	6,794	27,206	25.0	1.36 × 10^–5^	4.75 × 10^–3^	*MYB88*	MYB DOMAIN PROTEIN 88
AT3G10030	42	789	5.3	751	27,206	2.8	4.44 × 10^–5^	1.55 × 10^–2^		
AT1G06180	102	789	12.9	2,422	27,206	8.9	8.38 × 10^–5^	2.93 × 10^–2^	*ATMYBLFGN, MYB13*	MYB DOMAIN PROTEIN 13
AT3G15210	179	789	22.7	4,758	27,206	17.5	9.38 × 10^–5^	3.28 × 10^–2^	*ERF4, RAP2.5*	RELATED TO AP2 5, ETHYLENE RESPONSIVE ELEMENT BINDING FACTOR 4
AT3G04070	231	789	29.3	6,448	27,206	23.7	1.49 × 10^–4^	5.20 × 10^–2^	*NAC047*	NAC DOMAIN CONTAINING PROTEIN 47
AT5G02320	97	789	12.3	2,334	27,206	8.6	2.04 × 10^–4^	7.11 × 10^–2^	*MYB3R5*	MYB DOMAIN PROTEIN 3R-5
AT5G58850	181	789	22.9	4,895	27,206	18.0	2.13 × 10^–4^	7.44 × 10^–2^	*MYB119*	MYB DOMAIN PROTEIN 119
AT1G28370	205	789	26.0	5,657	27,206	20.8	2.21 × 10^–4^	7.72 × 10^–2^	*ERF11*	ERF DOMAIN PROTEIN 11
AT5G25190	161	789	20.4	4,281	27,206	15.7	2.38 × 10^–4^	8.32 × 10^–2^	*ESE3*	ETHYLENE AND SALT INDUCIBLE 3
AT5G65130	76	789	9.6	1,742	27,206	6.4	2.54 × 10^–4^	8.87 × 10^–2^		
AT2G42430	31	789	3.9	540	27,206	2.0	2.75 × 10^–4^	9.60 × 10^–2^	*ASL18, LBD16*	LATERAL ORGAN BOUNDARIES-DOMAIN 16, ASYMMETRIC LEAVES2-LIKE 18

*^*a*^The number of genes bound by the specific TF in the test set. ^*b*^The number of genes in the test set. ^*c*^The number of genes bound by the specific TF in the reference set. ^*d*^The number of genes in the reference set. ^*e*^The *p*-value after Bonferroni or Benjamini-Hochberg correction.*

**TABLE 4:** Summary statistics of enriched upstream TFs for down-regulated genes in *Arabidopsis* roots upon 1μM IAA treatment for 6 h (Omelyanchuk et al., 2017).

**TF ID (AGI ID)**	***x*^a^**	***n*^b^**	**Observed (%)**	***X*^c^**	***N*^d^**	**Expected (%)**	***p*-Value**	**Corrected *p*-value^e^**	**Gene symbols**	**Gene names**
AT3G62420	238	659	36.1	5,764	27,206	21.2	3.78 × 10^–19^	1.32 × 10^–16^	*BZIP53*	BASIC REGION/LEUCINE ZIPPER MOTIF 53
AT4G34590	289	659	43.9	7,781	27,206	28.6	2.33 × 10^–17^	8.12 × 10^–15^	*BZIP11, GBF6, bZIP11, ATB2*	G-BOX BINDING FACTOR 6, BASIC LEUCINE-ZIPPER 11
AT5G65310	451	659	68.4	14,295	27,206	52.5	3.52 × 10^–17^	1.23 × 10^–14^	*ATHB5*,	HOMEOBOX PROTEIN 5
AT4G36740	460	659	69.8	14,742	27,206	54.2	8.29 × 10^–17^	2.89 × 10^–14^	*HB-5, ATHB40*	HOMEOBOX PROTEIN 40
AT5G66700	283	659	42.9	7,658	27,206	28.1	1.44 × 10^–16^	5.03 × 10^–14^	*HB-8, ATHB53*	HOMEOBOX-8, HOMEOBOX 53
AT5G03790	381	659	57.8	11,605	27,206	42.7	1.77 × 10^–15^	6.17 × 10^–13^	*ATHB51, LMI1*	HOMEOBOX 51, LATE MERISTEM IDENTITY1
AT5G15830	244	659	37.0	6,440	27,206	23.7	5.41 × 10^–15^	1.89 × 10^–12^	*bZIP3*	BASIC LEUCINE-ZIPPER 3
AT1G14687	393	659	59.6	12,176	27,206	44.8	6.05 × 10^–15^	2.11 × 10^–12^	*HB32, ZHD14*	HOMEOBOX PROTEIN 32, ZINC FINGER HOMEODOMAIN 14
AT3G56850	169	659	25.6	3,936	27,206	14.5	1.84 × 10^–14^	6.42 × 10^–12^	*AREB3, DPBF3*	ABA-RESPONSIVE ELEMENT BINDING PROTEIN 3
AT1G69780	422	659	64.0	13,486	27,206	49.6	2.64 × 10^–14^	9.22 × 10^–12^	*ATHB13*	
AT1G12630	228	659	34.6	5,960	27,206	21.9	2.79 × 10^–14^	9.72 × 10^–12^		
AT1G32150	254	659	38.5	6,979	27,206	25.7	1.30 × 10^–13^	4.53 × 10^–11^	*bZIP68*	BASIC REGION/LEUCINE ZIPPER TRANSCRIPTION FACTOR 68
AT2G18550	229	659	34.7	6,124	27,206	22.5	2.91 × 10^–13^	1.01 × 10^–10^	*ATHB21, HB-2*	HOMEOBOX-2, HOMEOBOX PROTEIN 21
AT3G50260	400	659	60.7	12,825	27,206	47.1	1.07 × 10^–12^	3.73 × 10^–10^	*DEAR1, ATERF#011, CEJ1*	COOPERATIVELY REGULATED BY ETHYLENE AND JASMONATE 1, DREB AND EAR MOTIF PROTEIN 1
AT2G18160	110	659	16.7	2,268	27,206	8.3	1.48 × 10^–12^	5.17 × 10^–10^	*bZIP2, FTM3, ATBZIP2, GBF5*	G-BOX BINDING FACTOR 5, BASIC LEUCINE-ZIPPER 2, FLORAL TRANSITION AT THE MERISTEM3
AT2G46270	201	659	30.5	5,255	27,206	19.3	2.40 × 10^–12^	8.38 × 10^–10^	*GBF3*	G-BOX BINDING FACTOR 3
AT5G52020	224	659	34.0	6,069	27,206	22.3	2.49 × 10^–12^	8.69 × 10^–10^		
AT4G16750	353	659	53.6	10,971	27,206	40.3	2.63 × 10^–12^	9.18 × 10^–10^		
AT1G75390	115	659	17.5	2,485	27,206	9.1	8.58 × 10^–12^	3.00 × 10^–9^	*bZIP44*	BASIC LEUCINE-ZIPPER 44
AT1G69010	328	659	49.8	10,132	27,206	37.2	2.20 × 10^–11^	7.69 × 10^–9^	*BIM2*	BES1-INTERACTING MYC-LIKE PROTEIN 2
AT2G36270	165	659	25.0	4,188	27,206	15.4	5.50 × 10^–11^	1.92 × 10^–8^	*ABI5, GIA1*	ABA INSENSITIVE 5, GROWTH-INSENSITIVITY TO ABA 1
AT5G51990	279	659	42.3	8,325	27,206	30.6	7.80 × 10^–11^	2.72 × 10^–8^	*DREB1D, CBF4*	C-REPEAT-BINDING FACTOR 4, DEHYDRATION-RESPONSIVE ELEMENT-BINDING PROTEIN 1D
AT5G25810	82	659	12.4	1,602	27,206	5.9	1.22 × 10^–10^	4.26 × 10^–8^	*TNY*	TINY
AT1G71450	310	659	47.0	9,574	27,206	35.2	1.60 × 10^–10^	5.60 × 10^–8^		
AT3G10800	77	659	11.7	1,469	27,206	5.4	1.62 × 10^–10^	5.64 × 10^–8^	*BZIP28*	
AT1G77200	175	659	26.6	4,646	27,206	17.1	4.32 × 10^–10^	1.51 × 10^–7^		
AT4G25480	158	659	24.0	4,064	27,206	14.9	4.46 × 10^–10^	1.56 × 10^–7^	*DREB1A, CBF3*	C-REPEAT BINDING FACTOR 3, DEHYDRATION RESPONSE ELEMENT B1A
AT2G31220	55	659	8.3	913	27,206	3.4	6.64 × 10^–10^	2.32 × 10^–7^		
AT3G28920	203	659	30.8	5,711	27,206	21.0	1.42 × 10^–9^	4.97 × 10^–7^	*ZHD9, AtHB34*	ZINC FINGER HOMEODOMAIN 9, HOMEOBOX PROTEIN 34
AT4G32730	246	659	37.3	7,322	27,206	26.9	2.20 × 10^–9^	7.67 × 10^–7^	*MYB3R1, PC-MYB1*	MYB DOMAIN PROTEIN 3R1, C-MYB-LIKE TRANSCRIPTION FACTOR 3R-1

*^*a*^The number of genes bound by the specific TF in the test set. ^*b*^The number of genes in the test set. ^*c*^The number of genes bound by the specific TF in the reference set. ^*d*^The number of genes in the reference set. ^*e*^The *p*-value after Bonferroni or Benjamini-Hochberg correction.*

**FIGURE 2:**
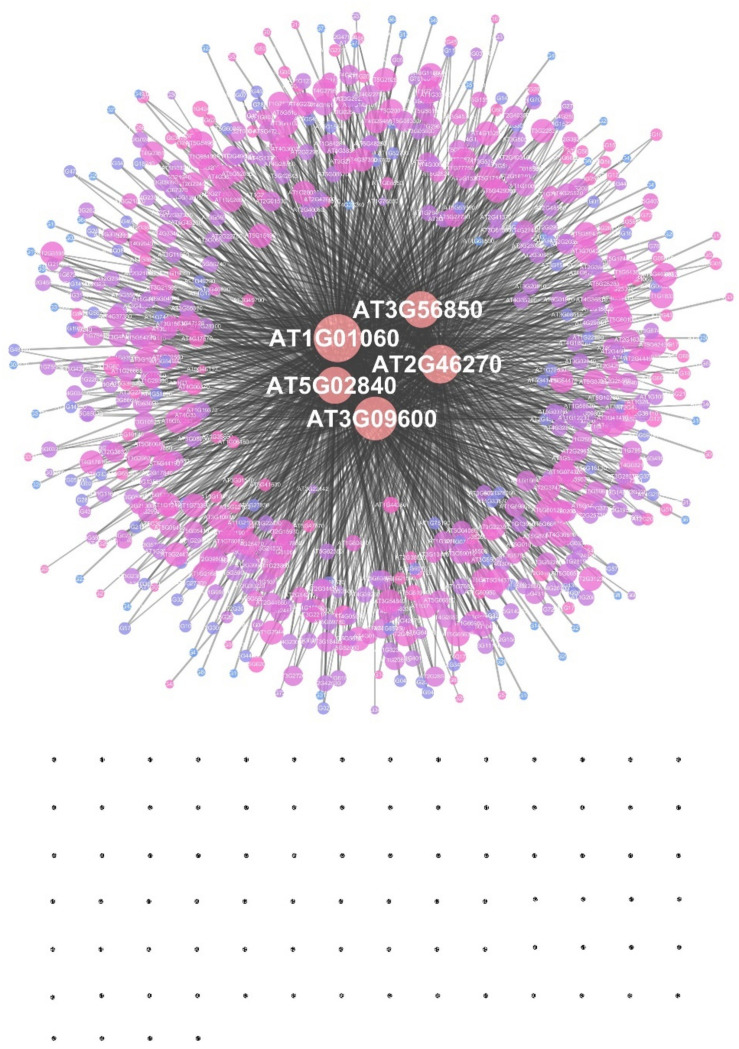
An example of a transcription factor regulatory network constructed by EAT-UpTF. A set of target genes of the LHY transcription factor (Adams et al., 2018) was used as a test input. The area of a node represents the edge count and the color intensity indicates the strength of the neighborhood connectivity. Black dots represent single nodes.

The EAT-UpTF analysis requires the input of an experimentally validated genome-wide list of TF-target genes in the form of locus ID. As mentioned above, we used the DAP-seq *Arabidopsis* database for the initial validation of EAT-UpTF. However, the EAT-UpTF analysis is not limited to the use of DAP-seq data and could also employ ChIP-seq data or any database that provides a list of TF-target genes. If only ‘bed’ files for DAP-seq and ChIP-seq are available, they can be converted to the EAT-upTF input format (Figure 1; see EAT-upTF homepage). In this regard, the EAT-UpTF analysis could be expanded to any plant species for which DAP-seq, ChIP-seq, or other appropriate sequencing data are available. In the future, a large-scale database integrating DAP-seq and ChIP-seq results would aid the identification of *bona fide* upstream TFs for groups of GOIs. EAT-UpTF is an open platform that can be improved by integrating updated TF databases. In addition, to ensure convenience for users, TF regulatory networks of GOIs identified by EAT-UpTF can also be visualized in Cytoscape (Figure 2). Compared to the previous webtools, such as TF2Network (Kulkarni et al., 2018) and AthaMap (Steffens et al., 2004), which conduct *cis*-element-based construction of TF regulatory networks, EAT-UpTF involves simple and rapid processing of data without *cis*-element identification, and thereby promptly visualizes gene regulatory networks showing TF-target gene interactions. While processing our study, a remarkable webtool ‘Plant Regulomics’^2^ has been released (Ran et al., 2020), which might implement a similar logic and code of EAT-UpTF, supporting the relevance of this analysis.

## Conclusion

In summary, EAT-UpTF is a tool for analyzing the over-representation of upstream TFs based on the relative enrichment of TF-target genes in a group of GOIs in plants. EAT-UpTF can be used to identify upstream TFs for a group of genes without limitations on species and conservation of *cis*-motifs. With a regular update or manual construction of databases of TF-target genes in plant species, EAT-UpTF will become a powerful tool for TF regulatory network studies in plants. For user convenience, EAT-UpTF web service is also available at http://chromatindynamics.snu.ac.kr:8080/EatupTF.

## Data Availability Statement

EAT-UpTF is available at https://github.com/sangreashim/EAT-UpTF; operating system(s): Linux, programming languages: Python3; other requirements: Python3 packages (SciPy, Statsmodels, Argparse). The EAT-UpTF home page provides detailed user manuals. EAT-UpTF is freely available. There are no restrictions on non-academics use.

## Author Contributions

SS and PS: conceptualization and funding acquisition. SS: data curation and implementation and writing – original draft. PS: writing – review and editing. Both authors contributed to the article and approved the submitted version.

## Conflict of Interest

The authors declare that the research was conducted in the absence of any commercial or financial relationships that could be construed as a potential conflict of interest.

## Footnotes

1 http://neomorph.salk.edu/dap_web/pages/index.php

2 http://bioinfo.sibs.ac.cn/plant-regulomics/
